# Identification of a novel phosphorylation site in hepatitis C virus NS5A

**DOI:** 10.1099/vir.0.023614-0

**Published:** 2010-10

**Authors:** Anna Nordle Gilliver, Stephen Griffin, Mark Harris

**Affiliations:** Institute of Molecular and Cellular Biology, Faculty of Biological Sciences, University of Leeds, Leeds LS2 9JT, UK

## Abstract

Hepatitis C virus (HCV) NS5A protein is phosphorylated on multiple residues; however, despite extensive study, the precise identity of these sites has not been determined unambiguously. In this study, we have used a combination of immunoprecipitation and mass spectrometry to identify these phosphorylation sites. This analysis revealed the presence of a major phosphorylated residue within NS5A from the genotype 1b Con1 isolate – serine 249 (serine 2221 in polyprotein numbering). However, mutation of this residue (or the corresponding threonine in the JFH-1 isolate) to either a phosphomimetic (aspartate) or a phosphoablative (alanine) residue resulted in no phenotype. We conclude that phosphorylation of this residue, in the context of a highly culture-adapted HCV genome, does not play a role in either viral RNA replication or virus assembly. It is possible that it might be important in an aspect of virus biology that is not recapitulated faithfully in the Huh-7 cell-culture system.

Hepatitis C virus (HCV) is estimated to infect some 123 million individuals (Shepard *et al.*, 2005), and establishes a chronic infection that can ultimately result in liver fibrosis, cirrhosis or hepatocellular carcinoma. Combination therapy comprising pegylated alpha interferon (IFN-*α*) and ribavirin is only successful in approximately 50 % of patients. HCV, a member of the family *Flaviviridae*, is an enveloped virus with a positive-sense RNA genome 9.6 kb in length and containing a single ORF. An internal ribosome entry site mediates cap-independent translation of the 3000 aa polyprotein, which is cleaved co- and post-translationally by host-cell and viral proteases to release the structural proteins (core, E1, E2 and p7) and non-structural proteins (NS2, NS3, NS4A, NS4B, NS5A and NS5B).

NS5A has been shown to have many functions: foremost, as a component of the RNA replication complex, it is absolutely required for viral RNA replication. Structural analysis has revealed that NS5A comprises three domains separated by short low-complexity regions (Tellinghuisen *et al.*, 2004) (Fig. 1, top). The structure of domain I has been determined; it coordinates a zinc ion and is postulated to dimerize (Love *et al.*, 2009; Tellinghuisen *et al.*, 2005). Domains II and III are less structured and more flexible; in particular, domain III can accommodate a GFP insert at the C terminus with no adverse effects (Appel *et al.*, 2005; McCormick *et al.*, 2006b; Moradpour *et al.*, 2004). By SDS-PAGE and Western blotting, two forms of the protein with different apparent mobilities can be observed: these correspond to alternatively phosphorylated forms of NS5A – a basally phosphorylated form (apparent molecular mass 56 kDa) and a hyperphosphorylated form (58 kDa). Proline-directed kinases such as casein kinase II (CKII) have been implicated in basal phosphorylation (Reed *et al.*, 1997), and CKI*α* has been implicated in hyperphosphorylation (Quintavalle *et al.*, 2007); however, there is little consensus as to either the locations or the number of phosphorylation sites on both forms of NS5A. Basal phosphorylation sites have been shown to be present in both domains II and III (Tanji *et al.*, 1995) and hyperphosphorylation sites have also been mapped to domain II (Katze *et al.*, 2000; Tanji *et al.*, 1995). Interestingly, inhibition of hyperphosphorylation either pharmacologically or by mutation enabled replication of a non-culture-adapted genotype 1b subgenomic replicon (Appel *et al.*, 2005; Neddermann *et al.*, 2004).

In order to assign phosphorylation sites unambiguously within NS5A, we adopted a mass-spectrometric (MS) approach. We showed previously that a recombinant baculovirus containing a tetracycline-responsive mammalian promoter (a BacMAM vector termed FBrepcon1neo; McCormick *et al.*, 2006a) could be used to drive high levels of expression of a Con1-derived genotype 1b NS3-5B subgenomic replicon in HepG2 cells. Importantly, HepG2 supported the replication of a subgenomic replicon delivered via the baculovirus route, as judged by induction of an IFN-*β* response that was not seen following delivery of a GND-mutant (replication-defective) replicon (McCormick *et al.*, 2004). Furthermore, NS5A expressed from this vector exhibited both basal and hyperphosphorylated species (p56 and p58) (McCormick *et al.*, 2006a), similar to those observed in Huh7 cells stably harbouring subgenomic replicons. HepG2 cells were therefore transduced with a BacMAM vector expressing the tetracycline transactivator (BactTA) together with FBrepcon1neo; NS5A was purified from cell lysates by using protein G–agarose and polyclonal sheep anti-NS5A serum, separated by SDS-PAGE and stained with colloidal blue stain. The p58 band was excised and submitted for MS analysis. Peptides from a trypsin digest were analysed on two systems independently: firstly, a 4000QTRAP system, where precursor ion scanning was used to identify phosphorylated peptides (Fingerprints Proteomics Facility, University of Dundee, UK) (Fig. 1), and secondly, a Waters Synapt HDMS system (Astbury Centre for Structural Molecular Biology, University of Leeds, UK) (data not shown). Potential phosphorylated peptides were then identified by MS/MS. In both cases, only one major phosphorylated peptide was identified. This was HDpSPDADLIEANLLWR [phospho-serine 249 (serine 2221 in polyprotein numbering)], which is situated at the junction of the first low-complexity sequence and domain II. This site has not been identified previously as a phosphorylation site, although it is situated close to a cluster of serine residues that have been postulated to be involved in hyperphosphorylation (serines 2197, 2201 and 2204) (Tanji *et al.*, 1995). There are a number of reasons why other phosphorylated peptides were not identified, including inefficient ionization, low stoichiometry and failure of some peptides to be captured by the ion trap. Therefore, the failure to identify other phosphorylation sites does not mean that the BacMAM-expressed NS5A in HepG2 cells is not phosphorylated on other residues. However, the identification of phospho-serine 249 by using two different systems in independent experiments suggests that this is a major site of phosphorylation within the hyperphosphorylated form of NS5A.

In order to assess the potential significance of phosphorylation at this residue for viral genomic replication, we generated two mutant forms of the FK5.1 culture-adapted subgenomic replicon (Krieger *et al.*, 2001) in which serine 249 was mutated to either an alanine (phosphoablative) or an aspartic acid (phosphomimetic) by PCR (oligonucleotide sequences are available upon request). We chose FK5.1 because the Con1 replicon is not culture-adapted and does not replicate at a sufficient level to allow discrimination between wild type and mutants with impaired replication efficiency. *In vitro* transcripts of the FK5.1 replicons were electroporated into Huh7 cells; cells were then selected for 3 weeks with 1 mg G418 ml^−1^, prior to either staining for colony-forming assays or generation of stable polyclonal replicon-harbouring cell lines. Neither mutation had any effect on either the number of colonies (Fig. 2a) or the p56 : p58 ratio (Fig. 2b). In order to exclude the possibility that the replicons had reverted to the wild-type sequence, RNA was extracted from cells, reverse-transcribed, amplified using PCR, cloned and sequenced (data not shown). All clones examined contained the original mutation and had thus not reverted to wild type.

These data were also confirmed in the context of a transient, luciferase-based FK5.1 subgenomic replicon. *In vitro* transcripts of the FK5.1 luciferase replicons were electroporated into Huh7 cells, and luciferase activity was measured at both 4 h post-transfection (to assess transfection efficiency and translation of input RNA) and 72 h (to assess RNA replication) (Fig. 2c). Relative replication levels for both mutants were similar to those of the wild type.

Serine 249 is almost completely conserved in all genotype 1 isolates of HCV; however, this residue is not conserved in other genotypes (Kuiken *et al.*, 2005). Notably, the equivalent residue in the only isolate that has the capacity for replication in cell culture, JFH-1, is threonine. In addition to this, a serine is situated two residues N-terminally (serine 247) in JFH-1 NS5A (Fig. 3a). The data in Fig. 2 demonstrated that phosphorylation of serine 249 was dispensable for RNA replication; therefore, to test whether the presence of a phosphorylatable residue at this position was important for the role of NS5A in virus assembly (Appel *et al.*, 2008; Hughes *et al.*, 2009; Masaki *et al.*, 2008; Tellinghuisen *et al.*, 2008a), we mutated either serine 247 or threonine 249 to either alanine or aspartic acid in the context of full-length JFH-1 virus (Wakita *et al.*, 2005), previously modified to contain synonymous mutations generating unique restriction sites flanking NS5A (Hughes *et al.*, 2009). Somewhat surprisingly, neither mutation had a substantial effect on either virus assembly or release (Fig. 3a). HCV protein synthesis in infected cells (Fig. 3b) and the NS5A p56 : p58 ratio were also unaffected. We conclude that phosphorylation of these residues is not important for either virus genome replication or particle assembly; however, we cannot rule out the possibility that, unlike Con1, in the context of FK5.1 or JFH-1 these residues are not phosphorylated. To test this, it would be necessary to express either the FK5.1 subgenomic replicon or full-length JFH-1 in HepG2 cells using a BacMAM vector and analyse the phosphorylation status of NS5A. Such experiments are under way in our laboratory.

What, then, might be the role of phosphorylation of serine 249? It is possible that, in the context of the culture-adapted FK5.1 subgenomic replicon or the highly efficiently replicating JFH-1 genome, there is no requirement for phosphorylation of this residue. However, in the context of the non-culture-adapted infectious clones (e.g. H77, J4, Con1), the phosphorylation of serine 249 might be important in some aspect of NS5A function. Indeed, the acquisition of culture-adaptive mutations in NS5A has been shown to correlate with a loss of hyperphosphorylation, even when the mutations were not at serine residues (Blight *et al.*, 2000). It will be of interest to investigate the potential role of serine 249 phosphorylation in the context of the infectious genotype 1b virus described recently (Pietschmann *et al.*, 2009). However, as acknowledged in that publication, the very low replicative capacity of this isolate in Huh7 cells will render such an investigation technically challenging. One attractive hypothesis that may be more tractable to investigation is the potential structural role of serine 249. As it is followed by a proline residue, phosphorylation of serine 249 might influence the recognition of this proline by peptidyl–prolyl isomerases such as cyclophilin A, a known NS5A-interacting partner that is important for viral RNA replication. The location of serine 249, precisely at the N terminus of domain II of NS5A, might allow phosphorylation to influence the spatial orientation of that domain with respect to domain I by mediating the *cis*–*trans* isomerization of the peptidyl–prolyl bond. Structural studies (e.g. NMR) on purified wild-type and mutant NS5A, phosphorylated *in vitro*, might shed light on this issue, but represent significant technical challenges.

## Figures and Tables

**Fig. 1.**
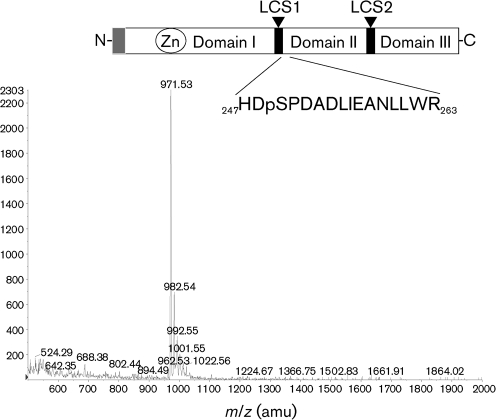
MS identification of serine 249 phosphorylation. At the top, a schematic of the structure of NS5A is depicted, showing the location of the phosphopeptide. LCS, Low-complexity sequence. For MS analysis, HepG2 cells were seeded onto rat tail collagen-coated plates and transduced with BactTA and FBrepcon1neo at 800 p.f.u. per cell each, as described previously (McCormick *et al.*, 2006a). Cells were lysed 24 h post-transduction and NS5A was purified by using protein G beads and a polyclonal sheep anti-NS5A serum (Macdonald *et al.*, 2003). Following SDS-PAGE, the colloidal blue-stained band corresponding to p58 was excised and digested with trypsin. The tryptic digests were analysed by liquid chromatography–MS with precursor ion scanning on a 4000QTRAP system. Sums of the peptide masses detected by precursor ion scanning in the negative-ion mode across the 45 min HPLC separation are shown. The peptide was identified from the MS/MS fragmentation spectra by database searching and manual inspection of the spectra. amu, Atomic mass units.

**Fig. 2.**
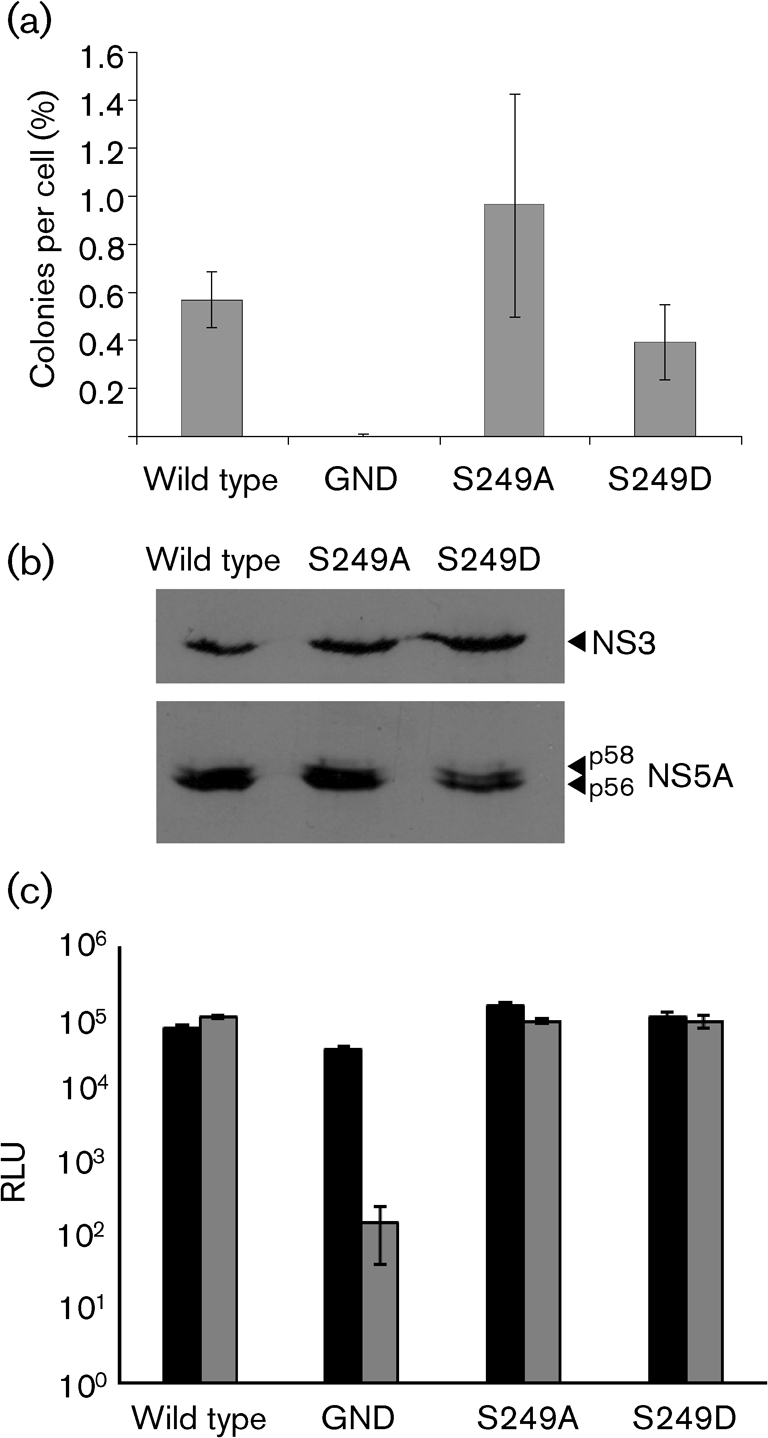
Role of serine 249 phosphorylation in genotype 1b RNA replication. (a) Huh7 cells (4×10^6^) were electroporated with the indicated *in vitro*-transcribed FK5.1 RNAs (2 μg), selected in the presence of G418 (1 mg ml^−1^) for 3 weeks and colonies were stained with Coomassie brilliant blue. Number of colonies produced is presented as a percentage of the initial number of electroporated cells. (b) Western blot analysis of lysates from stable cell lines harbouring the indicated FK5.1 replicons with sheep polyclonal antiserum to either NS3 (Aoubala *et al.*, 2001) or NS5A (Macdonald *et al.*, 2003). (c) Huh7 cells were electroporated with replicon RNA and harvested into passive lysis buffer (Promega) at the indicated time points (black bars, 4 h; grey bars, 72 h). Luciferase activity (relative luciferase units; RLU) was measured as described previously (Macdonald *et al.*, 2003). In both graphs, error bars show sem; data from three independent experiments are shown.

**Fig. 3.**
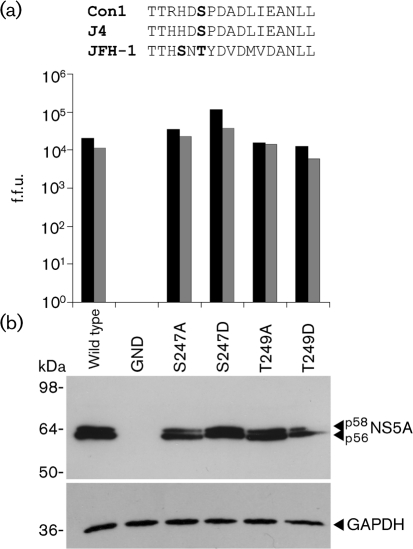
Role of serine 247/threonine 249 phosphorylation in assembly and release of infectious HCV. (a) Sequence of the region surrounding serine 249 in Con1, J4 and JFH-1 NS5A. Mutated residues are shown in bold. Huh7 cells (4×10^6^) were electroporated with the indicated *in vitro*-transcribed virus RNAs (10 μg) and virus release into the culture supernatant (black bars) was measured by focus-forming assay (Hughes *et al.*, 2009). Intracellular virus titres (grey bars) were measured by focus-forming assay following cell disruption by repetitive freeze–thaw at 72 h post-transfection (p.t.). (b) Huh7 cells were electroporated with the indicated virus RNAs and harvested at 48 h p.t. by lysis in Glasgow lysis buffer (Harris & Coates, 1993). Protein (10 μg) was analysed by Western blotting with antiserum to NS5A or glyceraldehyde-3-phosphate dehydrogenase (GAPDH) as indicated.
